# Multi-omics integration identifies regulatory factors underlying bovine subclinical mastitis

**DOI:** 10.1186/s40104-024-00996-8

**Published:** 2024-03-14

**Authors:** Mengqi Wang, Naisu Yang, Mario Laterrière, David Gagné, Faith Omonijo, Eveline M. Ibeagha-Awemu

**Affiliations:** 1 Agriculture and Agri-Food Canada, Sherbrooke Research and Development Centre, Sherbrooke, QC Canada; 2 Department of Animal Science, Université Laval, Quebec City, QC Canada; 3 Quebec Research and Development Centre, Agriculture and Agri-Food Canada, Quebec City, QC Canada

**Keywords:** Candidate discriminant multi-omics signature, Gene, Long non-coding RNA, Methylation haplotype block, MicroRNA, Multi-omics integration, Natural killer cell mediated cytotoxicity pathway, *Staphylococcus aureus* infection pathway

## Abstract

**Background:**

Mastitis caused by multiple factors remains one of the most common and costly disease of the dairy industry. Multi-omics approaches enable the comprehensive investigation of the complex interactions between multiple layers of information to provide a more holistic view of disease pathogenesis. Therefore, this study investigated the genomic and epigenomic signatures and the possible regulatory mechanisms underlying subclinical mastitis by integrating RNA sequencing data (mRNA and lncRNA), small RNA sequencing data (miRNA) and DNA methylation sequencing data of milk somatic cells from 10 healthy cows and 20 cows with naturally occurring subclinical mastitis caused by *Staphylococcus aureus* or *Staphylococcus chromogenes*.

**Results:**

Functional investigation of the data sets through gene set analysis uncovered 3458 biological process GO terms and 170 KEGG pathways with altered activities during subclinical mastitis, provided further insights into subclinical mastitis and revealed the involvement of multi-omics signatures in the altered immune responses and impaired mammary gland productivity during subclinical mastitis. The abundant genomic and epigenomic signatures with significant alterations related to subclinical mastitis were observed, including 30,846, 2552, 1276 and 57 differential methylation haplotype blocks (dMHBs), differentially expressed genes (DEGs), lncRNAs (DELs) and miRNAs (DEMs), respectively. Next, 5 factors presenting the principal variation of differential multi-omics signatures were identified. The important roles of Factor 1 (DEG, DEM and DEL) and Factor 2 (dMHB and DEM), in the regulation of immune defense and impaired mammary gland functions during subclinical mastitis were revealed. Each of the omics within Factors 1 and 2 explained about 20% of the source of variation in subclinical mastitis. Also, networks of important functional gene sets with the involvement of multi-omics signatures were demonstrated, which contributed to a comprehensive view of the possible regulatory mechanisms underlying subclinical mastitis. Furthermore, multi-omics integration enabled the association of the epigenomic regulatory factors (dMHBs, DELs and DEMs) of altered genes in important pathways, such as ‘*Staphylococcus aureus* infection pathway’ and ‘natural killer cell mediated cytotoxicity pathway’, etc., which provides further insights into mastitis regulatory mechanisms. Moreover, few multi-omics signatures (14 dMHBs, 25 DEGs, 18 DELs and 5 DEMs) were identified as candidate discriminant signatures with capacity of distinguishing subclinical mastitis cows from healthy cows.

**Conclusion:**

The integration of genomic and epigenomic data by multi-omics approaches in this study provided a better understanding of the molecular mechanisms underlying subclinical mastitis and identified multi-omics candidate discriminant signatures for subclinical mastitis, which may ultimately lead to the development of more effective mastitis control and management strategies.

**Supplementary Information:**

The online version contains supplementary material available at 10.1186/s40104-024-00996-8.

## Introduction

Bovine mastitis, an inflammatory disease of the mammary gland, is a major concern for the dairy industry because it leads to significant economic impacts through reduced milk production, lower milk quality, high veterinary costs, early culling of infected cows, animal welfare issues and reproductive problems, amongst others [1, 2]. Mastitis is a multi-factorial disease caused by various types of bacteria, viruses and other pathogens, and occurs in both clinical and subclinical forms. Subclinical mastitis is characterized by long term infection without visible signs in the mammary gland or milk [3] but an increase in milk somatic cell count (SCC), which indicates an immune response to the infection [4]. A range of bacteria could cause subclinical mastitis, however, *Staphylococcus aureus* and coagulase-negative staphylococci, such as *Staphylococcus chromogenes*, are considered the primary pathogens because of the negative economic impact they have on dairy production [5]. Cows with subclinical mastitis are at an increased risk of developing clinical mastitis, which may cause even more severe symptoms and discomfort, and lead to higher veterinary costs and even early culling. Therefore, the development of effective mastitis management practices is essential to the success and sustainability of the dairy industry.

The rapid development of cutting-edge sequencing technologies has provided valuable insights into the molecular mechanisms of mastitis, which may contribute to the improvement of breeding programs and the development of new strategies for managing and controlling mastitis. For instance, genome-wide association studies (GWAS) have identified numerous genetic variations related to milk production and health of dairy cows [6–8]. These variations have been utilized in genomic breeding programs, contributing significantly to the achievement of higher genetic gains in genomic breeding programs and in the improvement of animal production, health and profitability in the dairy industry [9–11]. Moreover, various studies profiled the transcriptome of different tissues, such as mammary gland, milk somatic cells and blood cells, and revealed important differentially expressed genes, gene networks, biological processes and pathways involved in the host immune response to subclinical mastitis [12–16]. In addition to genetic mechanisms, studies of epigenetic modifications, including DNA methylation, non-coding RNAs and histone modifications have furthered understanding of the regulatory mechanisms underlying bovine subclinical mastitis [17–19]. For example, DNA methylation alterations have been found to mediate the gene expression changes in response to mastitis, which provided further insights into the molecular mechanisms of the phenotype variations unexplained by genetic factors [20, 21].

These single omics studies, including genomics, transcriptomics and methylomes amongst others, have made important contributions to our understanding of bovine mastitis. However, they provided a limited view of mastitis by focusing on single layers of biological information and may not provide systematic functional information, which is necessary for understanding the biological mechanisms underlying mastitis pathogenesis. To address these limitations, the multi-omics approach has emerged as a promising tool for gaining deeper understanding and elucidation of the potential causative molecular mechanisms that underlie subclinical mastitis. Applying the multi-omics approach to integrate a range of high dimensional datasets at multiple layers could reveal new interactions or relationships among different biological processes, thereby contributing to providing a more complete view of a biological system [22, 23]. The multi-omics approach has been applied to various fields, including human disease, medicine, agriculture and environmental science, to gain a better understanding of complex biological systems and to develop new diagnostic and therapeutic approaches [24, 25]. However, it is important to note that the application of multi-omics approaches in studying bovine mastitis is still in its early stages. For instance, a few studies integrated two omics data, such as GWAS and RNA-sequencing data [26], transcriptome and miRNA profiles [27–29], or transcriptome and methylome [30–33], and revealed novel insights into the genetic and epigenetic basis of mastitis. This highlights the importance of using multi-omics approaches to gain a deeper understanding of mastitis and emphasizes the advantage of integrating more omics data for a more comprehensive investigation of the pathogenesis and regulatory mechanisms of mastitis.

In light of the complex and multifaceted nature of subclinical mastitis, our study aims to provide a more comprehensive understanding of its molecular mechanisms. Through the integration of multiple omics data from milk somatic cells, including mRNA transcriptome, DNA methylome, and non-coding RNA (ncRNA) transcriptomes (specifically, long non-coding RNA (lncRNA) and microRNA (miRNA)), we seek to delineate a multi-omics network (DNA methylation-ncRNA-mRNA) elucidating the hub pathways involved in the pathogenesis of subclinical mastitis. Furthermore, our objective is to identify a set of candidate discriminant signatures with the potential to serve as biomarkers in mastitis control strategies. By achieving these goals, we aspire to significantly contribute to the augmentation of our current understanding of the intricate regulatory systems governing subclinical mastitis. Ultimately, we anticipate that our study’s outcomes will offer valuable insights for the development of novel strategies to control mastitis, encompassing interventions such as diagnostics, treatments, and enhance prediction of breeding values in breeding programs.

## Materials and methods

### Animals and sample collection

Lactating dairy cows with subclinical mastitis from commercial dairy farms in Quebec, Canada, were recruited for the study. Further detailed information regarding cow selection and sample collection procedures can be found in our previous reports [15, 32]. In brief, we firstly monitored the milk SCC records of all lactating cows for a period of 6 months using Fossomatic flow cytometric cell counter (Lactanet, Sainte-Anne-de-Bellevue, Quebec, https://lactanet.ca/). Cows with consecutively high (> 350,000 cells/mL) (potential subclinical mastitic (SM) cows) (*n* = 81) or low SCC (< 100,000 cells/mL) (potential heathy control (HC) cows) (*n* = 63) for a period of 3 or more months were selected for the study. Then 5 mL milk samples from each quarter of SM cows and a 20 mL composite sample from each HC cow were collected and sent to Biovet laboratories (St-Hyacinthe, QC, Canada) for bacteriological examination. Fifteen HC cows that tested negative for mastitis pathogens were kept as the HC group. Among cows of SM group, eighteen tested positive for *Staphylococcus aureus* (SAP) and four cows tested positive for *Staphylococcus chromogenes* (SCP) in at least one quarter, and were enrolled as SM group (*n* = 22). Next, we collected about 200 mL composite milk samples from HC cows (equal volumes from all four quarters), and 200 mL milk sample from one positive quarter (even if more than one quarters were positive for a pathogen) of SM cows. Following collection, a small part of each milk sample (~ 2 mL) was sent for bacteriological examination to validate pathogen infection identified in the first bacteriological results. Meanwhile, milk somatic cells were isolated from the remaining milk samples by low speed centrifugation (1500 × *g*, 15 min, 4 °C). Finally, we kept milk somatic cell samples from 10 HC and 20 SM cows (comprising 16 SAP cows and 4 SCP cows) for next-step analysis, which had consistent bacteriological examination results and enough milk somatic cells.

Animal use procedures were approved in accordance with the guidelines of the Canadian Council on Animal Care, and ethical approval to conduct the study was provided by the Animal Care and Ethics Committee of Agriculture and Agri-Food Canada (approval #570).

### Generation of multi-omics data

The milk somatic cells per cow were divided into two equal parts, one part was used for the isolation of DNA and the other part to which an equal volume of Trizol reagent was added, was used for the isolation of total RNA using respectively DNeasy Blood and Tissue Kit, and RNeasy Mini Kit (Qiagen Inc., Toronto, ON, Canada). The genomic DNA was used for whole genome-wide DNA methylation sequencing (WGMS), while the total RNA was subjected to RNA sequencing for characterization of mRNA and lncRNA transcriptomes, and small RNA sequencing for miRNA profiling. The details regarding the generation of multi-omics data, including DNA methylation and mRNA transcriptome and miRNA expression profiles are found in our previous reports [15, 32, 34, 35]. Briefly, the library preparation and deep sequencing were performed by Centre d'expertise et de services Génome Québec (Canada). We used standard bioinformatics pipelines to process the raw WGMS reads, RNA sequencing reads (mRNA and lncRNA) and miRNA sequencing reads [36]. Following sequence quality check, the clean sequences were mapped to the bovine reference genome ARS-UCD1.2 [37]. Next, the method of methylation haplotype blocks (MHBs) was used to represent the DNA methylation status. The MHBs were identified using MONOD2 [38] and defined as regions that harbored at least 3 CpG sites and in which the adjacent CpG sites were associated (linkage disequilibrium *r*^2^ ≥ 0.5). The MHBs were firstly identified for SM-SAP and SM-SCP data sets separately as described in our previous studies [32, 34]. For this study, we kept the common MHBs identified in both data sets to represent the DNA methylation omics. Then, the methylated haplotype load (MHL), which is a weighted mean of the fraction of methylated methylation haplotypes at different lengths, were used to compare the methylation status of MHBs between SM and HC groups by using two-tailed Student’s *t*-test to identify differential MHBs (dMHBs). The *P*-value was adjusted by Benjamini and Hochberg false discovery rate (FDR) method [39]. Significant dMHBs were defined as MHBs with ≥ 20% difference in MHL and FDR > 0.05. From the RNA sequencing data, the mRNA and lncRNA profiles were identified meanwhile the miRNAs were identified from the small RNA sequencing data. For the transcriptome data (mRNA, miRNA and lncRNA), we firstly filtered to remove those with low reads (low expression level). We kept genes (mRNAs), lncRNAs and miRNAs with ≥ 10 reads count in ≥ 50% of samples per group for the next-step analysis. We then used DESeq2 for the normalization of read counts and identification of differentially expressed genes (DEGs), lncRNAs (DEL) and miRNAs (DEM) between SM and HC groups, where FDR < 0.05 and |log_2_ fold change (log_2_FC)|≥ 1 were used as thresholds to define significant differences between SM and HC groups.

### Integrative single sample gene-set analysis of multi-omics data

Firstly, we used the multi-omics gene-set analysis R package program, MOGSA [40], to integrate multiple omics datasets, including genes (mRNAs), MHBs, lncRNAs and miRNAs, and annotated the multi-omics features to functional gene sets. MOGSA is an enrichment approach that relies on matrix factorization of multi-omics data measured on the same samples, which is powerful to learn patterns of biological significance in high dimensional data [40]. MOGSA generates gene set scores (GSSs) for each sample by learning the most variant features following integration of all input omics. Since MOGSA does not require pre-filtering of data but requires multi-omics data measured on the same samples, we used all expressed mRNAs, lncRNAs, miRNAs and the identified MHBs in milk somatic cells from 24 samples which had data on the four omics, including nineteen SM cows (15 SAP and 4 SCP cows) and 5 HC cows. The default parameters were used in the analysis. A total of 321 KEGG (Kyoto Encyclopedia of Genes and Genomes) pathways and 7654 Gene Ontology (GO) biological processes (BP-GO) terms were downloaded from KEGG [41] and GO [42] databases, respectively, and used as functional gene sets. The significant gene sets were defined as having significant GSS (FDR < 0.05) in more than two thirds of the samples (*n* = 16).

### Discovery of the principal sources of variation in multi-omics data

Secondly, we performed Multi-Omics Factor Analysis (MOFA) to discover the principal sources of variation in the four omics data sets by using MOFA2 [43]. The expression level (read count) of genes, lncRNAs and miRNAs were normalized for size factor normalization and variance stabilization with DESeq2. The DNA methylation data (MHL of MHBs) was normalized by using the quantile normalization method. The normalized omics datasets (DEG, DEL, DEM and dMHB) were inputted into MOFA2 (version 1.8.0) for integrative analysis. MOFA2 uses a probabilistic approach to model the shared and dataset-specific factors that underlie the variability across the multiple omics datasets. The identified factors can then be used to identify the molecular signatures and pathways that are associated with each factor. The default number of factors (*n* = 10) was used which was trimmed based on the minimum variance explained criteria (2%). The model was firstly trained by MOFA2 function ‘prepare_mofa’ and then used for factor analysis by using the MOFA2 function ‘run_mofa’, and the identified factors were interpreted based on the features with the highest factor loadings.

The results of the factor analysis were visualized using the MOFA2 functions. For instance, heatmaps were plotted to display the variance decomposition by factor and the total variance explained per omics. While the factor values and feature weights were exhibited by scatterplot. The get set enrichment analysis was then performed on the identified factors by using the MOFA2 function “run_enrichment”. The KEGG pathways and BP-GO terms were used as the gene set annotations. The statistical significance of the identified pathways and BP-GO terms was determined using a FDR threshold of 0.05.

### Identification of candidate discriminant signatures from multi-omics data

In order to identify highly correlated multi-omics signatures that discriminate the SM (SAP and SCP) and HC groups, we used the DIABLO (Data Integration Analysis for Biomarker discovery using a Latent component method for Omics studies) method implemented in the R package, mixOmics version 6.22.0 [44], to integrate the four omics datasets. The input data for DIABLO included normalized expression values of DEGs, DELs, DEMs and dMHBs (describe in data preparation of MOFA2) from 19 SM cows (15 SAP and 4 SCP cows) and 5 HC cows which had all four omics data. Before using the DIABLO framework, we examined the correlation between omics in a non-integrative context by using pairwise PLS (Projection to Latent Structure) comparisons that revealed strong correlation between the omics data sets. To initialize the DIABLO model, we used a weighted design (0.1) to achieve the balance between maximizing the correlation between omics datasets and maximizing the discriminative ability of the model. The optimal number of components and number of signatures to choose per omics were detected by using the function ‘perf’ and ‘tune.block.splsda’, respectively, using fourfold cross-validation repeated 10 times. The final DIABLO model with optimal parameters was run with function ‘block.splada’ to identify the candidate discriminant signatures, including genes, miRNA, lncRNAs and MHBs. The results were visualized with functions from mixOmics, including diagnostic plot (‘plotDiablo’)—shows the correlation between each input omics, circus plot (‘circosPlot’)—displays the correlation between identified signatures, bar plot (‘plotLoadings’)—presents the loading weight per signature per omics on each component, and clustered image map (‘cimDIABLO’)—shows the multi-omics signature expression per sample.

### Target gene prediction, functional annotation and visualization

TargetScan8.0 [45] was used to predict the target genes of miRNAs by using thresholds of weighted context++ score < −0.2 and weighted context++ score percentile ≥ 95. The target genes of miRNAs which were not expressed in the mRNA transcriptome of milk somatic cells were removed. In addition, we further checked the possible correlations between miRNAs and their predicted target genes by using Spearman's rank correlation coefficient with the R package, Hmisc version 5.0–1 [46]. Only the predicted target genes that were also significantly negatively correlated with the corresponding miRNA (*P* < 0.05 and *r* < −0.3) were kept as target genes of miRNAs for next-step analysis. To prepare the gene set annotation file for miRNA, the involvement of a miRNA in a gene set (BP-GO term or KEGG pathway) was defined as the miRNA that targets at least one gene in the corresponding gene set. Besides, the target genes of lncRNAs were predicted by their *cis-* and *trans-*role. The *cis*-target genes of lncRNAs were identified by co-location of protein-coding genes (mRNA) in the 100 kb upstream and downstream regions of the corresponding lncRNA and also expressed in milk somatic cells. Then we calculated the Spearman's rank correlation coefficient (*r*) in one-to-one correspondence between detected genes and lncRNAs using Hmisc [46]. The genes with a *P* < 0.01 and |*r*|> 0.95 with a lncRNA were filtered as the *trans*-target (co-expressed) genes of the corresponding lncRNA. Similarly, the involvement of a lncRNA in a gene set was defined as the lncRNA that *cis*-/*trans*-targets at least one gene of the corresponding gene set.

## Results

### Multi-omics data collection

**Fig. 1:**
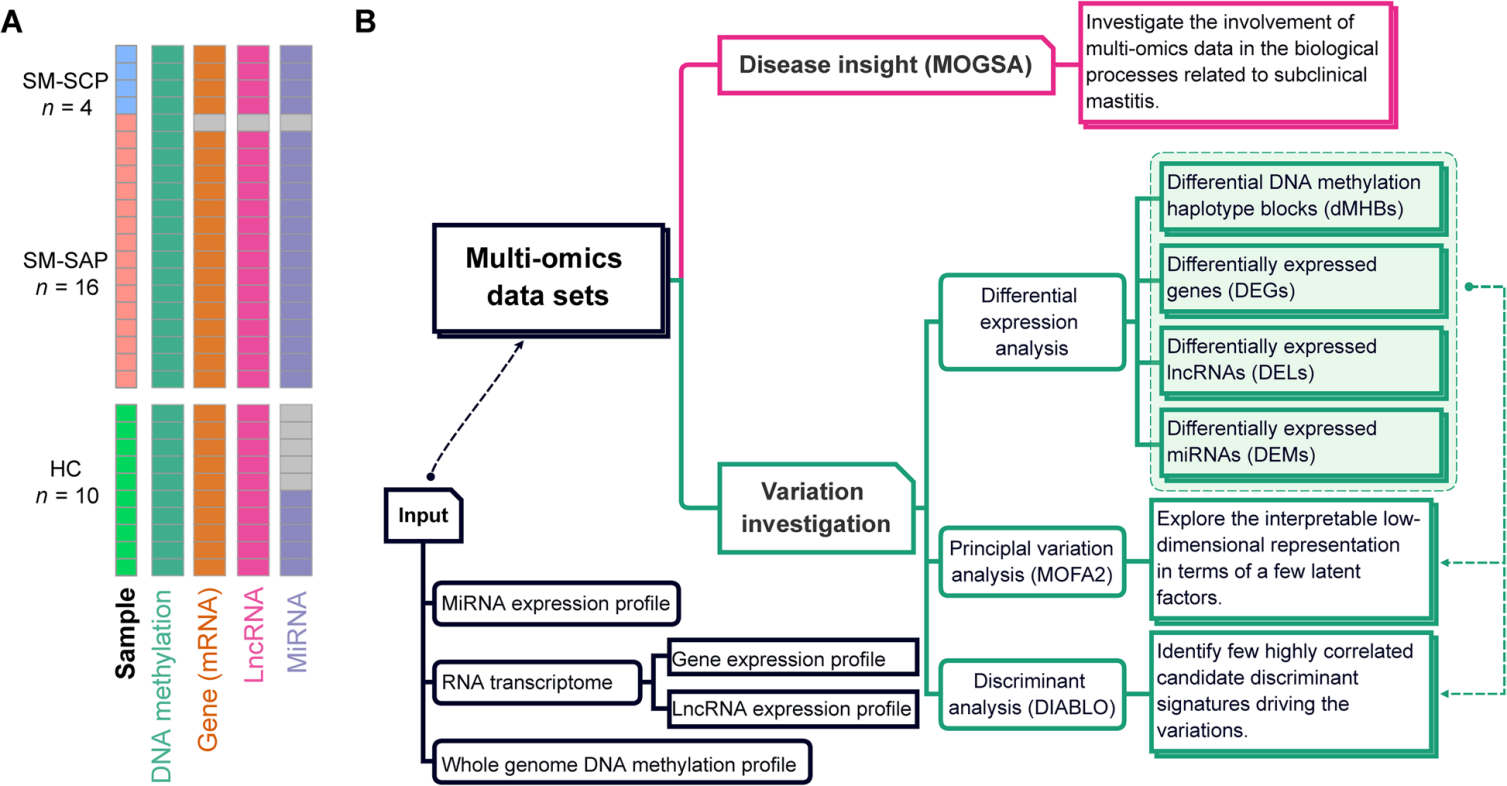
Overview of sample type (**A**) and workflow of multi-omics approaches (**B**). **A** Overview of sample type and availability for each omics. The number of samples are displayed as rows and omics data sets as columns. SM-SCP: Subclinical mastitis due to *Staphylococcus chromogenes*, SM-SAP: Subclinical mastitis due to *Staphylococcus aureus*, HC: Healthy control, MOGSA: Multi-omics gene-set analysis, MOFA2: Multi-Omics Factor Analysis, DIABLO: Data integration analysis for biomarker discovery using latent variable approaches for omics studies

Milk somatic cells from 30 cows, including 20 SM (16 SAP and 4 SCP) and 10 HC cows were used to construct the multi-omics profiles responding to subclinical mastitis (Fig. 1A). The WGMS data was available for all 30 cows, however, one cow (SM-SAP) lacked RNA sequencing data and 6 cows (1 SM-SAP and 5 HC cows) lacked small RNA sequencing data due to limited sample volumes. Finally, a total of 30 DNA methylation profiles, 29 transcriptomes including gene (mRNA) and lncRNA profiles and 24 miRNA profiles of milk somatic cells were used (Fig. 1A). We identified a total of 55,854 MHBs from WGMS sequences, 13,581 mRNAs and 3183 lncRNAs from RNA sequences, and 288 miRNAs from small RNA sequences, which were leveraged to derive insights into mastitis biology and identify important genetic and epigenetic signatures (Fig. 1B).

### Biological insight of subclinical mastitis by multi-omics data integration

**Fig. 2:**
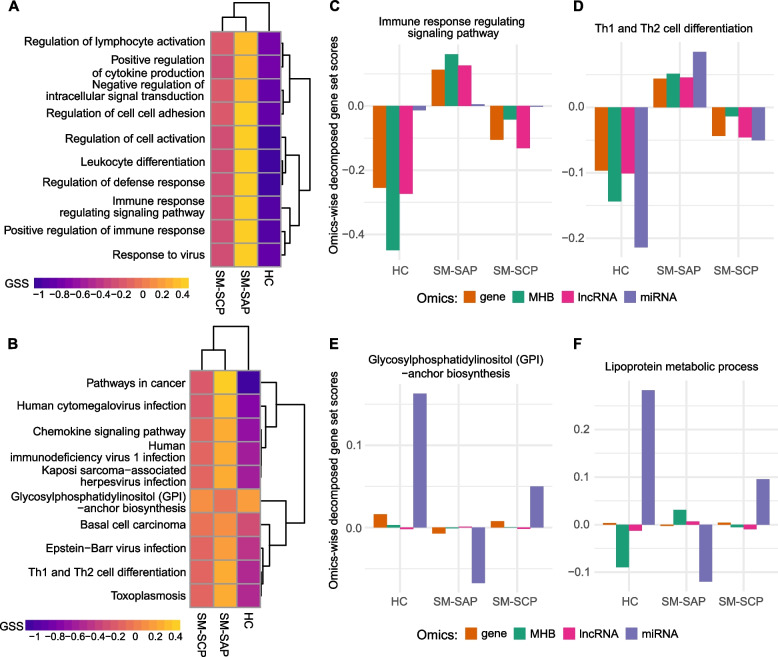
Integrative gene set analysis. **A** and** B** Heatmaps showing the gene set scores (GSSs) of the top 10 most significantly regulated GO terms (**A**) and KEGG pathways (**B**) in milk somatic cells. **C** and **D** Omics-wise decomposition of the GSS for some of the gene sets. The contribution of each omics is represented by the corresponding bar, and the *y*-axis means the omics-wise decomposed GSS. SM-SCP: subclinical mastitis due to *Staphylococcus chromogenes*, SM-SAP: subclinical mastitis due to *Staphylococcus aureus*, HC: health control, MHB: methylation haplotype block

Next, we used the multi-omics signatures to derive further insights into mastitis biology by using MOGSA. Due to no missing value allowed for MOGSA, 24 samples with data from all four omics (5 HC and 19 SM cows) were used. MOGSA identified 3458 BP-GO terms (out of 7773) with significant up- or down-regulated GSSs in at least 16 samples (FDR < 0.05). The majority of the significant BP-GO terms (*n* = 2458, 70.84%) had higher GSSs in SM group compared to HC group, with most of them being immune functions or disease related terms. For example, the most significant BP-GO term, “regulation of cell activation”, had significant GSS in all 24 samples (i.e., large difference of GSS between SM and HC groups) (Fig. 2A). Next, 244 GO terms had significant GSS in 23 samples, most of which showed large difference of GSSs between SM and HC groups, such as “leukocyte differentiation”, “regulation of defense response”, “immune response regulating signaling pathway”, “positive regulation of immune response”, “response to virus” “regulation of cell cell adhesion” and “regulation of lymphocyte activation”, etc. (Fig. 2A, Table S1). In contrast, the BP-GO terms with lower GSSs in SM group are mostly related to metabolic processes, transmembrane transport and other biosynthesis related processes (Table S1). Moreover, the MOGSA also identified 170 significant KEGG pathways, including 134 and 36 KEGG pathways with higher and lower GSSs in SM group compared to HC group, respectively. Consistent with BP-GO terms, most of the KEGG pathways with up-regulated GSSs were disease pathways or pathways with important immune functions, such as “Th1 and Th2 cell differentiation”, “Pathway in cancer”, “Chemokine signaling pathway”, “Th17 cell differentiation” and “Human cytomegalovirus infection” amongst others (Fig. 2B, Table S1). Meanwhile, the KEGG pathways with down-regulated GSSs are related to biosynthesis and metabolism, such as “Glycosylphosphatidylinositol (GPI)-anchor biosynthesis”, “Beta-Alanine metabolism” and “Arginine biosynthesis”. It is worth noting that GSS difference between SM and HC groups were generally greater in immune/disease-related KEGG pathways than the metabolism-related KEGG pathways, suggesting that the altered activities of immune functions were stronger than metabolism processes during subclinical mastitis.

We further introduced the data-wise decomposition of the GSSs to evaluate the relative contribution (either concordant or discrepant) of each omics to the overall GSSs. As shown in Fig. 2C and D, the four omics had concordant contributions to most of BP-GO terms and KEGG pathways within group but different contributions between groups. For instance, most immune related BP-GO and KEGG pathways, such as “immune response regulating signaling pathway” (Fig. 2C), “Th1 and Th2 cell differentiation” (Fig. 2D), “positive regulation of immune response”, “Chemokine signaling pathway”, and “NF-kappa B signaling pathway” amongst others, had significant positive GSS from all four omics in SM-SAP group, but negative GSS in HC and SM-SCP groups (Table S1). This suggests the up-regulated activities of immune-related processes in SM-SAP group. However, we also observed discrepant contributions in the four omics to the GSS of some metabolism/biosynthesis related BP-GO terms and KEGG pathways. For example, mRNA and miRNA had negative GSS in SM-SAP group but positive GSS in HC and SM-SCP groups for the KEGG pathway “Glycosylphosphatidylinositol (GPI)-anchor biosynthesis” (Fig. 2E). However, lncRNA showed opposite contribution to the GSS of this term (“Glycosylphosphatidylinositol (GPI)-anchor biosynthesis”) by being positive in SM-SAP group but negative in HC and SM-SCP groups. Another case is the BP-GO term “lipoprotein metabolic process”, to which MHB and lncRNA contributed positively to the GSS but mRNA and miRNA showed negative GSS for this term in SM-SAP group, which is opposite for SM-SCP and HC groups (Fig. 2F). Interestingly, the overall GSS in SM-SCP group to this term (“lipoprotein metabolic process”) is similar to that of SM-SAP group, however, the decomposed contribution of the four omics to the GSS was similar to HC group. This is consistent with the characteristics of SM-SCP, in that SC causes very mild mastitis. Thus the decomposition of GSS evaluated the contribution of each omics data to the gene sets, revealing their involvements in the altered immune responses and impaired mammary gland productivity during subclinical mastitis.

### Genomic and epigenomic alterations in response to subclinical mastitis

**Fig. 3:**
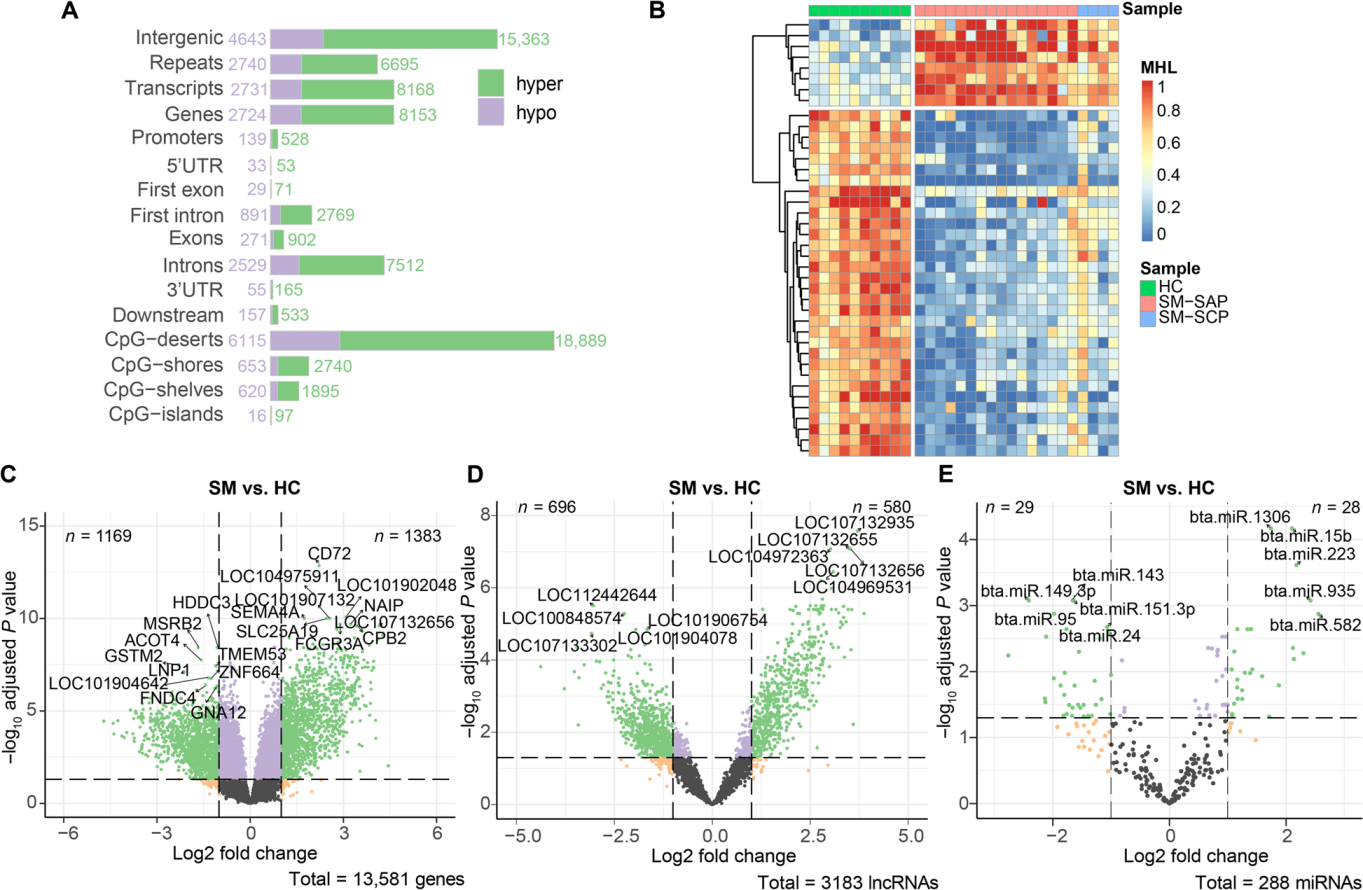
The Genomic and epigenomic alterations in response to subclinical mastitis (SM). **A** Distribution of differential methylation haplotype blocks (dMHBs) in genomic regions. Hyper and hypo represents the hyper- and hypo-methylation of dMHB in SM group compared to healthy control (HC) group. **B** Heat map of the top 40 most significant dMHBs showing the clear clustering of cows from SM and HC groups. **C**–**E** Volcano plots showing the differentially expressed genes, lncRNAs, and miRNAs. The number of up- and down-regulated signatures are noted on the top right and left corners of corresponding plots

We next identified the multi-omics alterations relevant to subclinical mastitis by comparing the SM cows to HC cows for each omics with all available data sets of the corresponding omics (Fig. 1A). In order to obtain the DNA methylation signatures related to SM caused by *S. aureus* or *S. chromogene*, we kept the 55,854 MHBs commonly identified in both SM-SAP and SM-SCP datasets to represent the DNA methylation omics. By comparing the methylation status of these MHBs in SM group to HC group, we found 30,846 differential MHBs (dMHBs) (|MHL difference| ≥ 20% and FDR < 0.05) (Table S2A). Among them, 76.16% (23,492) showed significantly higher methylation levels in SM cows compared to HC cows, while only 7354 (23.84%) were hypo-methylated in SM cows. The majority of dMHBs are located in the intergenic regions or CpG deserts (Fig. 3A), followed by genes and repeat elements. Within genes, most dMHBs were collocated with introns. We also identified 4365 dMHBs that were overlapped with regulatory regions, including promoter regions (*n* = 667), first exons (*n* = 100) and first introns (3660). It is worth noting that dMHBs, particularly the most variable ones, showed clear differences and clustering between SM and HC cows (Fig. 3B). A total of 13,581 genes (mRNAs) with at least 10 read counts in more than 50% of cows per group were used as the mRNA transcriptome omics. In addition, we identified 2552 differentially expressed genes (DEGs) (|log_2_FC| ≥ 1 and FDR < 0.05), including 1383 up-regulated and 1169 down-regulated DEGs, by comparing the transcriptome data of SM cows to HC cows (Fig. 3C, Table S2B). The top 10 most significant DEGs were all up-regulated in SM cows, including *CD72* (CD72 molecule), *LOC104975911*, *LOC101902048*, *LOC101907132*, *SLC25A19* (solute carrier family 25 member 19), *SEMA4A* (semaphorin 4A), *NAIP* (NLR family apoptosis inhibitory protein), *FCGR3A* (Fc gamma receptor IIIa), *LOC107132656*, and *CPB2* (carboxypeptidase B2) (Fig. 3C). Meanwhile, we also mined 3183 lncRNAs from the RNA-Seq transcriptome data as lncRNA omics, and identified 1276 differentially expressed lncRNAs (DELs) (|log_2_FC| ≥ 1 and FDR < 0.05), including 696 down-regulated and 580 up-regulated DELs in SM group compared to HC group (Fig. 3D, Table S2C). LOC112442644 and LOC107132935 were the most significant down- and up-regulated DEL, respectively (Fig. 3D). Furthermore, a total of 288 known miRNAs with at least 10 read counts in at least 50% of the samples per group were kept to represent the miRNA omics. We identified 57 differentially expressed miRNAs (DEMs), comprising 28 up-regulated and 29 down-regulated DEMs in SM cows compared to HC cows (Fig. 3E, Table S2D). The top 10 most significant DEMs included 5 up-regulated DEMs (bta.miR.15b, bta-miR-1306, bta-miR-223, bta-miR-935 and bta-miR-582) and 5 down-regulated DEMs (bta-miR-151-3p, bta-miR-143, bta-miR-149-3p, bta-miR-95, bta-miR-24), and the top 3 are all up-regulated, including bta-miR-15b, bta-miR-1306, and bta-miR-223 (Fig. 3E).

### The multi-omics principal variance related to subclinical mastitis

**Fig. 4:**
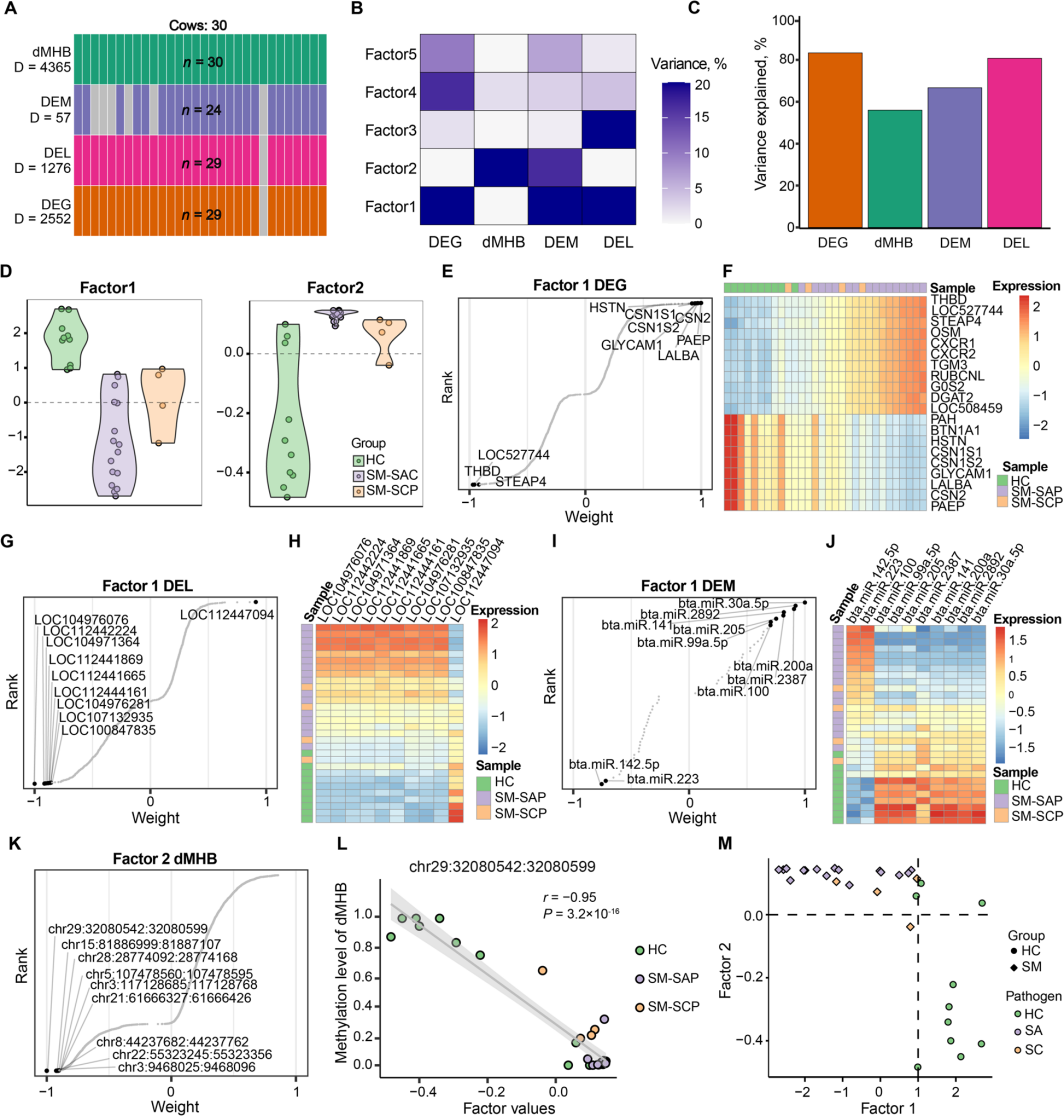
Multi-Omics Factor Analysis (MOFA) of subclinical mastitis. **A** Overview of samples and data type. Data modalities are showed in different rows (D represents the number of signatures) and samples are displayed in columns where the missing samples were marked in grey color. **B** The proportion of total variance explained per factor per omics. **C** The cumulative proportion of total variance explained by each omics. **D** Factor values of Factors 1 and 2 colored by the health condition, including healthy control (HC), subclinical mastitis due to *Staphylococcus aureus* (SM-SAP) and *Staphylococcus chromogenes* (SM-SCP). **E**, **G**, **I** Feature weights of differentially expressed genes (DEGs) (**E**), lncRNAs (DELs) (**G**), and miRNAs (DEMs) (**I**) associated with Factor 1. **F**, **H**, **J** Heatmaps showing the expression values of the top 20 DEGs (**F**), top 10 DELs (**H**), and DEMs (**J**) with greatest absolute weights in Factor 1. **K** Feature weights of differential methylation haplotype blocks (dMHBs) associated with Factor 2. **L** The Factor 2 values versus methylation level of dMHB chr29:32,080,542:32,080,599, which had largest absolute weight (negative). **M** Visualization/grouping of samples using Factors 1 and 2. The various shapes and colors represents samples according to group and pathogen

The significant differential signatures from the four omics, including 4365 dMHBs overlapping with regulatory regions, 2552 DEGs, 1276 DELs and 57 DEMs were input data for MOFA2 for the principal variation analysis. Notably, MOFA2 was designed to cope with missing values, therefore all 30 samples were used (Fig. 4A). MOFA2 identified 5 factors which explain at least 2% of the variance in at least one omics (Fig. 4B, Table S3A). Among them, Factors 1 and 2 captured most of the variance, suggesting broad roles in the trait under investigation. Factor 1 captured the variance that is present across DEG, DEM and DEL. Meanwhile, Factor 2 captured the co-variance between dMHB and DEM. On the contrary, Factor 3 was very active in a single omics capturing strong variance in DEL and to a lesser extent in DEG. Factor 4 captured a strong source of variance in DEG and weak variance in 3 omics, and Factor 5 was slightly active and capture weak variance in DEG, DEM and DEL. Cumulatively, the 5 factors explained about 80% variance in DEG (82.82%) and DEL (81.00%), 55.98% variance of dMHB and 66.55% variance of DEM (Fig. 4C).

As showed in Fig. 4D, Factor 1 had positive factor values in HC group and negative factor values in most SM samples. This indicate that the signatures, including DEG, DEM and DEL, positively associated with Factor 1 (positive weight) had higher expression levels in HC group (down-regulated in SM group compared to HC group). While the signatures with negative weights had up-regulated expression in SM group compared to HC group. Based on the top weights, a large number of signatures were identified with association with Factor 1 (Fig. 4E–J, Table S3B–D). Notably, more up-regulated DEGs (with negative weights) had greater absolute weights than the down-regulated DEGs (with positive weight), revealing the more important contribution (stronger association) of up-regulated DEGs to Factor 1. For example, amongst the most important DEGs with largest absolute weights, *PAEP* (progestagen-associated endometrial protein), *CSN2* (casein beta), *LALBA* (lactalbumin alpha), *GLYCAM1* (glycosylation dependent cell adhesion molecule 1), *CSN1S2* (casein alpha-S2), *CSN1S1* (casein alpha-S1), and *HSTN* (histatherin) had down-regulated expression in SM group while *THBD* (thrombomodulin), *LOC527744*, and *STEAP4* (STEAP4 metalloreductase) had up-regulated expression (Fig. 4E, F). Interestingly, the most up-regulated DEGs such as *CXCR1* (chemokine (C-X-C motif) receptor 1), *CXCR2* (C-X-C motif chemokine receptor 2), *TGM3* (transglutaminase 3), *THBD* and *STEAP4*, etc., have immune-related functions (Fig. 4F). Similarly, most of the important DELs with greater absolute weights were negatively associated with Factor 1 (negative weight) and up-regulated in SM group compared to HC group, such as LOC104976076, LOC112441114, and LOC104971364, etc. (Fig. 4G). Only one lncRNA (LOC112447094) in the top 10 most important DEL had higher expression level in HC group (Fig. 4H). However, more DEMs had greater weights and were down-regulated in SM groups (Fig. 4I and J). For instance, bta-miRNA-30-5p had the greatest absolute weight, suggesting the strongest association with Factor 1, but was significantly down-regulated in SM groups (log_2_FC = −1.86). Besides, a high portion of variance in dMHB was captured by Factor 2 which had positive factor value in most SM cows and negative factor value in most HC cows (Fig. 4D). The majority of dMHBs with strong association with Factor 2 (|weight| > 0.5) were hypo-methylated (negative weight) in SM group compared to HC group (Fig. 4K). For example, the most important dMHB with largest absolute weight was chr29:32,080,542:32,080,599, which was nearly fully methylated in HC group but barely methylated in SM group (Fig. 4L). As shown in Fig. 4B and Fig. 4D–L, both Factors 1 and 2 are associated with the difference in the mammary gland health condition (SM or HC), which captures the significant variance of multi-omics signatures. Interestingly and importantly, the combination of Factors 1 and 2 classified the cows into subgroups depending on the molecular profile of the four input omics, which was capable to separating SM cows from HC cows (Fig. 4 M).

**Fig. 5:**
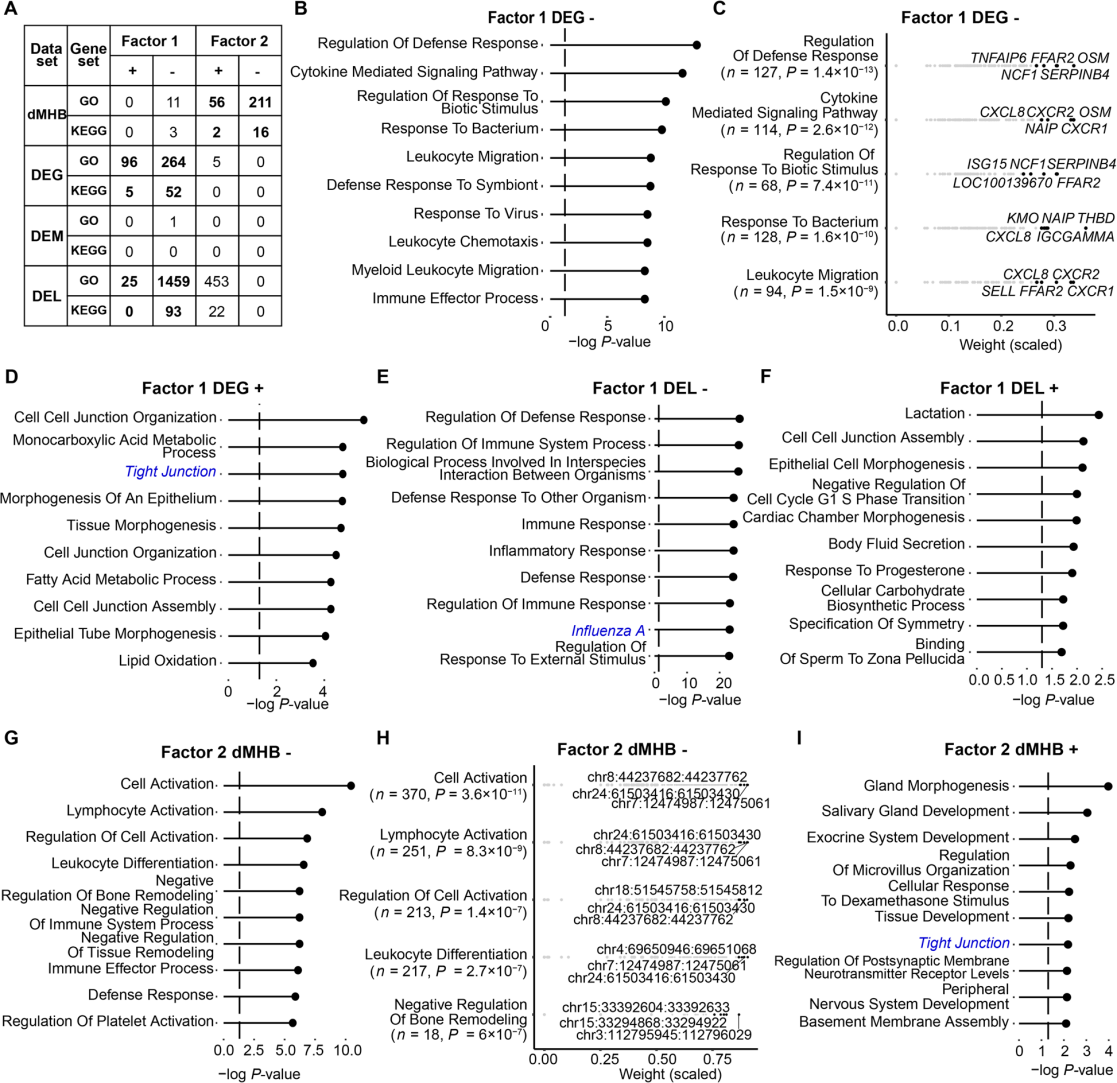
Gene sets enriched for by Factors 1 and 2. **A** Number of enriched gene sets, including KEGG pathways and biological process GO terms, per omics of Factors 1 and 2. “+” and “-” mean the signatures with positive (+) and negative (−) weights in corresponding factors. **B**, **D**–**G**, **I** The top 10 most significant gene sets enriched for by DEGs with negative (**B)** and positive weights (**D**) in Factor 1, DELs with negative (**E**) and positive weights (**F**) in Factor 1, and dMHBs with negative (**G**) and positive weights (**I**) in Factor 2. The KEGG pathways are marked in italic with blue color, while the GO terms are in regular font and black color. **C** and **H** Details of top 5 significant gene sets enriched for by DEGs with negative weights in Factor 1 (**C**) and dMHBs with negative weights in Factor 2 (**H**) where *n* and *P* represent the number of involved signatures and *P* value of the corresponding gene set. GO: Gene Ontology; DEG: Differentially expressed gene; DEL: Differentially expressed lncRNA; DEM: Differentially expressed miRNA; dMHB: Differential methylation haplotype block

In addition to identifying associated signatures per factor by individual weights discussed above, we also investigated the possible roles of Factors 1 and 2 by using gene set enrichment analysis (Fig. 5A, Table S4A–D). The DEGs with negative weights (up-regulated expression) in Factor 1 were enriched in 264 BP-GO terms and 52 KEGG pathways, which are mostly related to immune functions and disease (Table S4A). For instance, the most significant gene sets are “regulation of defense response”, “cytokine mediated signaling pathway”, “regulation of response to biotic stimulus”, “response to bacterium”, “leukocyte migration” and others (Fig. 5B and C). Meanwhile, the DEGs with positive weight representing down-regulated expression in SM group were enriched in less gene sets, including 96 BP-GO and 5 KEGG pathways, which are mostly related to metabolic processes and cell morphology (Fig. 5D). Similarly, the DEL with negative and positive weights in Factor 1 were significantly enriched in gene sets related to immune functions and morphogenesis/biosynthesis processes, respectively (Fig. 5E and F, Table S4B). This is consistent with the up-regulated activities of immune related gene sets and down-regulated activities of gene sets related to cellular activities and metabolic processes detected by MOGSA, revealing the detailed involvement of genomic and epigenomic signatures in the mammary gland responses to subclinical mastitis.

Moreover, the dMHBs negatively correlated with Factor 2 (hypo-methylated) were significantly enriched in gene sets, predominant of which were related to immune functions, such as “cell activation”, “lymphocyte activation”, “regulation of cell activation”, “leukocyte differentiation”, and others (Fig. 5G–H, Table S4C). Most of these gene sets were also enriched for by up-regulated DEGs and DELs in Factor 1. Meanwhile, the dMHBs positively associated with Factor 2 (hyper-methylated) were significantly enriched in gene sets which are important for developmental processes, such as “gland morphogenesis”, “salivary gland development” and “exocrine system development” and others (Fig. 5I).

**Fig. 6:**
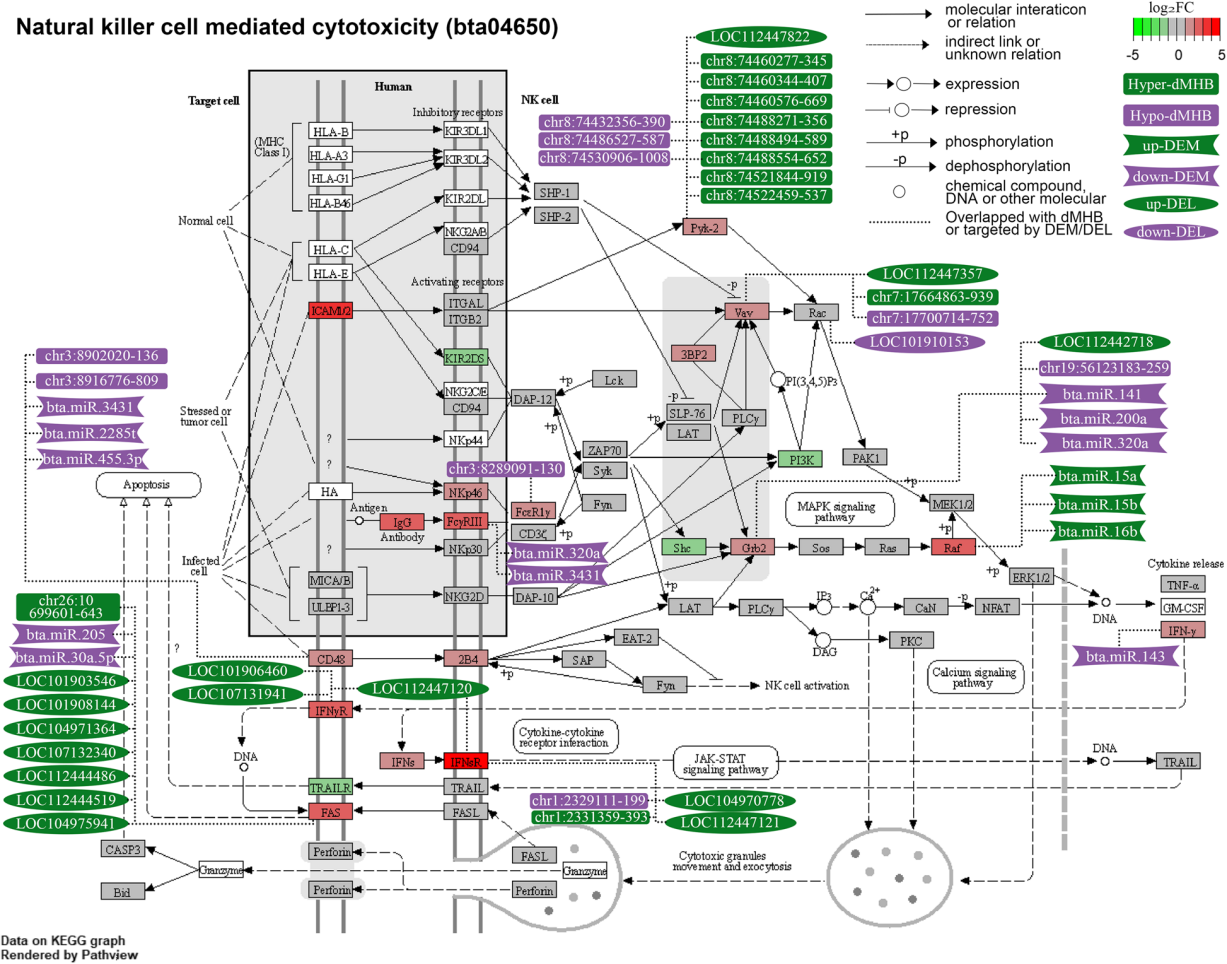
Network of multi-omics signatures involved in “natural killer cell mediated cytotoxicity” pathway during subclinical mastitis. Log_2_FC (Log_2_ Fold Change) is the mRNA expression level changes of corresponding genes (shown in square box) in subclinical mastitis group compared to healthy control group. Hyper(hypo)-dMHB: hyper-/hypo-methylated differential methylation haplotype blocks; up(down)-DEM: up-/down-regulated differentially expressed miRNA; up(down)-DEL: up-/down-regulated differentially expressed lncRNA. The comparison of multi-omics signatures between groups were processed by comparing subclinical mastitis group to healthy control group

Intriguingly, we observed the involvement of multi-omics signatures associated with Factors 1 and 2. For instance, the involvement of upregulated DEGs and DELs negatively correlated with Factor 1 and hypo-methylated dMHBs negatively correlated with Factor 2 were found in 78 gene sets, including 72 GO terms and 6 KEGG pathways, most of which were related to immune functions (Table S4E). Moreover, the significant altered activities of 64 out of these 78 gene sets (60 GO terms and 4 KEGG pathways) were also detected by MOGSA, further suggesting the integration of multi-omics signatures in the regulation of important biological processes during subclinical mastitis. For example, Fig. 6 showed the multi-omics signatures involved in the KEGG pathway “Natural killer cell mediated cytotoxicity”. In addition to DEGs, dMHBs, DELs and DEMs possibly regulating the corresponding DEGs, were demonstrated, suggesting that the DNA methylation and non-coding RNAs may serve as the regulatory mechanisms governing the gene expression alterations, thereby affecting the activities of natural killer cell mediated cytotoxicity during subclinical mastitis. Amongst them, the up-regulated *CD48* harbored 2 hypo-methylated dMHBs (chr3:8,902,020–8,902,136 and chr3:8,916,776:8,916,809) at its regulatory region and is targeted by 3 down-regulated DEMs (bta-miR-3431, bta-miR-2285t, and bta-miR-455-3p), which is consistent with the classic repressive effects of DNA methylation and miRNAs on the expression of corresponding gene. Similarly, the up-regulated *FCGR3A* and *IFNG* (interferon gamma) were targeted by down-regulated DEMs (bta-miR-320a and bta-miR-3431 target *FCGR3A* and bta-miR-143 targets *IFNG*, and up-regulated *FCER1G* (Fc epsilon receptor Ig) harbored hypo-methylated chr3:8,289,091:8,289,130 at its first exon. Besides, we also found the hyper-methylated dMHBs and up-regulated DELs associated with the corresponding DEGs, such as the presence of hyper-methylated dMHBs at the regulatory region of up-regulated *PYK2* (protein tyrosine kinase 2 beta) and multiple up-regulated DELs target up-regulated *FAS* (Fas cell surface death receptor). This suggests other possible effects of DNA methylation and lncRNAs on the gene expression which deserves further investigation.

**Fig. 7:**
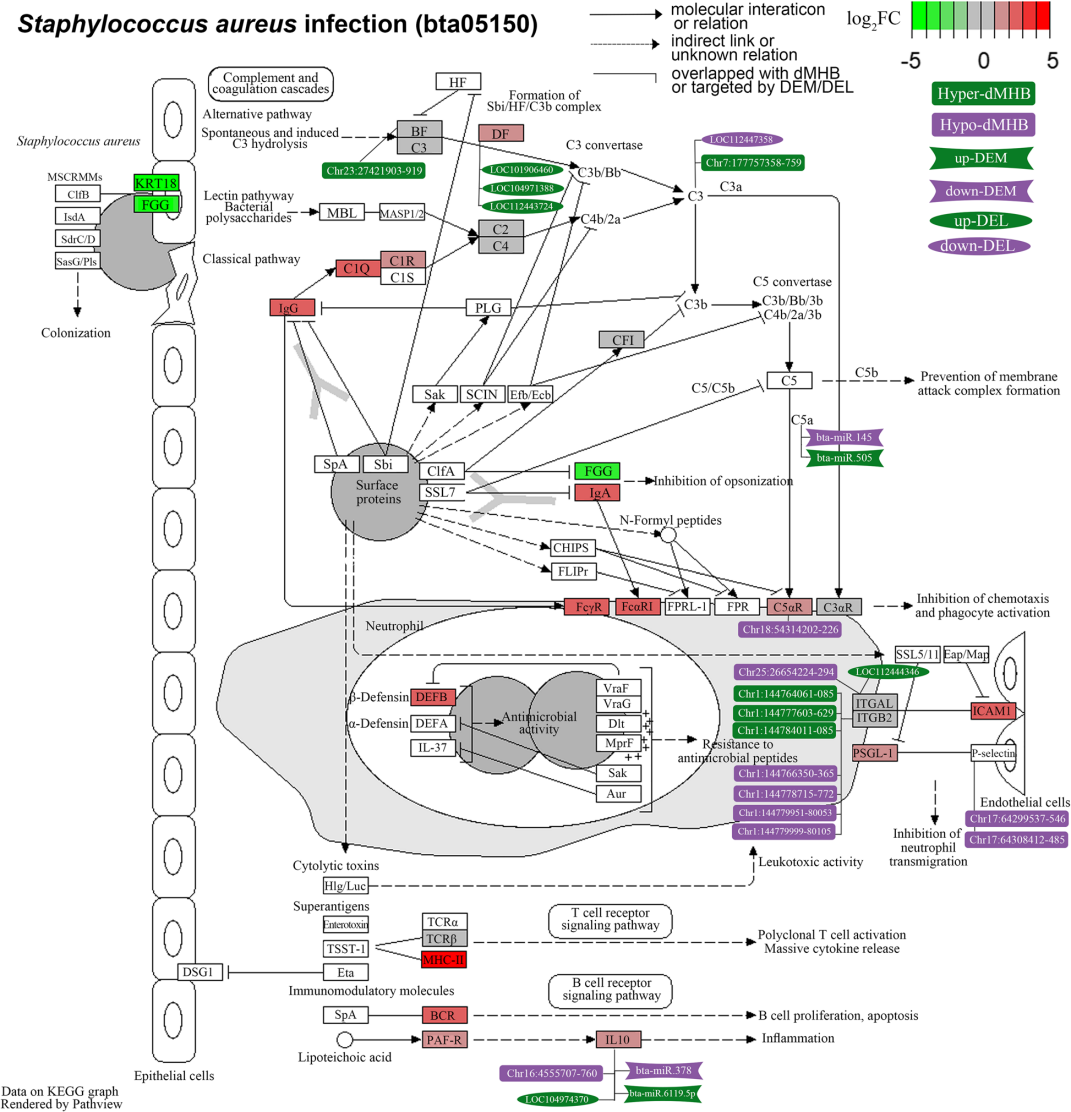
Network of multi-omics signatures involved in “*Staphylococcus aureus* infection” pathway during subclinical mastitis. Log_2_FC (Log_2_ Fold Change) is the mRNA expression level changes of corresponding genes (shown in square box) in subclinical mastitis group compared to healthy control group. Hyper(hypo)-dMHB: hyper-/hypo-methylated differential methylation haplotype blocks, up(down)-DEM: up-/down-regulated differentially expressed miRNA, up(down)-DEL: up-/down-regulated differentially expressed lncRNA. The comparison of multi-omics signatures between groups were processed by comparing subclinical mastitis group to healthy control group

Furthermore, the involvement of multi-omics signatures has also been observed in the “*Staphylococcus aureus* infection” (Fig. 7), a crucial pathway for subclinical mastitis. This pathway was significantly enriched by up-regulated DEGs negatively associated with Factor 1, where dMHBs, DELs, and DEMs are also involved in. Notably, the up-regulated *IL10* (interleukin 10), an important gene for inflammation harbored a hypo-methylated dMHB (chr16:4,555,707:4,555,760) at its promoter region. *IL10* was also found to be predictably targeted by an up-regulated DEL (LOC104974370), an up-regulated DEM (bta-miR-6119-5p) and a down-regulated DEM (bta-miR-378). This provided possible post-transcriptional mechanisms governing the expression alteration of *IL10* during subclinical mastitis. Similarly, a hypo-methylated dMHB (chr18:54,314,202:54,314,226) was found in the first intron of *C5αR1* (complement C5*α* receptor 1), which play roles in the inhibition of chemotaxis and phagocyte activation. Besides, 3 up-regulated DELs, including LOC10906460, LOC104971388 and LOC112443724, were observed to target the up-regulated *DF*. Although significant differences where not found in the expression levels of *ITGAL* (integrin subunit alpha L) and *ITGB2* (integrin subunit beta 2) between SM and HC groups, many dMHBs were found in their regulatory regions, which may play roles in regulating the host response to subclinical mastitis in other ways. The identified multi-omics signatures could provide a more comprehensive view of the *S. aureus* infection processes of the mammary gland, which may contribute to the development of related control strategies.

In summary, the up-regulated DEG, DEL and DEM associated with Factor 1 and the hypo-methylated dMHB associated with Factor 2 were crucial for the immune functional processes, while the down-regulated DEG, DEL and DEM and hyper-methylated dMHBs were more involved in the metabolic and developmental processes. This is consistent with the known repressive effects of DNA methylation on transcriptional activities. This also suggests that the multi-omics signatures associated with Factors 1 and 2 may provide a better network for understanding the regulatory mechanisms underlying subclinical mastitis.

### Discriminant signatures across omics for subclinical mastitis

**Fig. 8:**
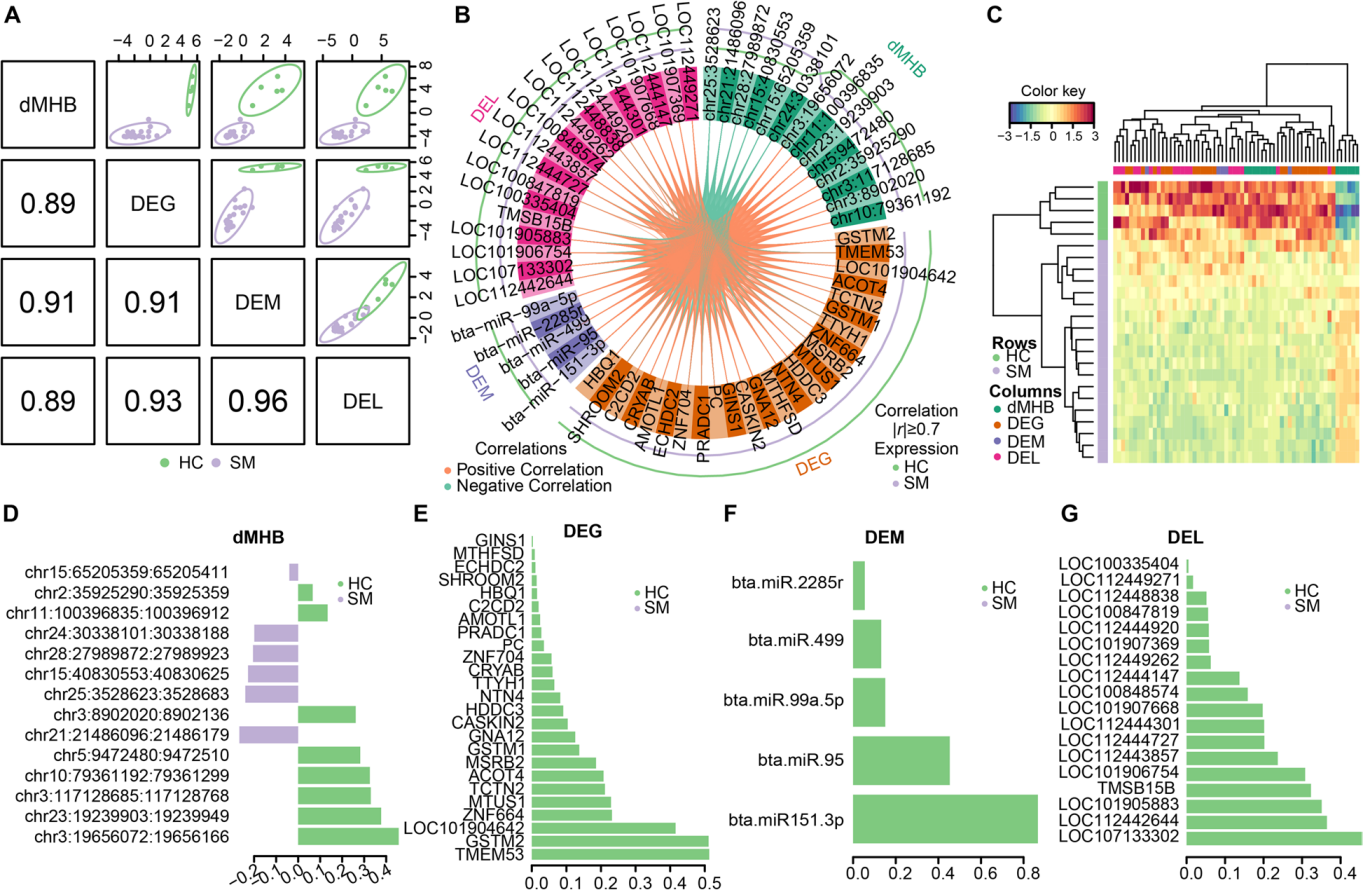
Identification of candidate discriminant signatures. **A** Diagnostic plot reveals that the first component from all omics datasets were highly correlated. **B** Circos plot of identified discriminant signatures per omics. The plot represents the correlations greater than 0.7 between signatures of different omics. The color of internal connecting lines shows the positive (orange) and negative (green) correlations. The outer lines represent the expression levels of corresponding signatures in SM and HC groups, with the outer line representing a higher expression in the corresponding group. **C** Clustered heat map for all identified candidate discriminant signatures. **D**–**G** The loading weight of identified signatures from the omics datasets of dMHB (**D**), DEG (**E**), DEM (**F**), and DEL (**G**). The most important discriminant signatures with highest absolute loading weights are ordered from bottom to top. The color of the bar indicates the group for which the median expression value is the highest for corresponding signatures. SM: Subclinical mastitis, HC: Healthy control, DEG: Differentially expressed gene, DEL: Differentially expressed lncRNA, DEM: Differentially expressed miRNA, dMHB: Differential methylation haplotype block

We also used DIABLO method to integrate the four omics and investigate their relationship with the mammary gland health condition (SM or HC), and to consequently identify a short list of multi-omics candidate discriminant signatures for subclinical mastitis. The differential multi-omics signatures, including 4365 dMHBs overlapping with regulatory regions, 2552 DEGs, 1276 DELs and 57 DMEs were loaded into DIABLO, and only the 24 samples with all four omics data were used. As shown in Fig. 8A, the strong correlation structure between the four omics data sets were extracted on the first components (*r* ≥ 0.89), and the SM and HC cows were clustered separately for each omics indicating the discriminative power of these components. A total of 14 dMHBs, 25 DEGs, 18 DELs and 5 DEMs were identified as the candidate discriminant signatures from each omics (Fig. 8B). The majority of the multi-omics candidate discriminant signatures were significantly strongly correlated with each other (Fig. 8B). We observed strong negative correlations (*r* < −0.7) between 6 hyper-methylated dMHBs with most of the other signatures, while the majority of strong positive correlations (*r* > 0.7) were observed between the other signatures (Fig. 8B). The average expression levels of identified discriminant signatures were significantly different, indicating they are able to discriminate the SM and HC groups (Fig. 8B and C).

Among the candidate discriminant dMHB signatures, 6 were hyper-methylated and 8 were hypo-methylated in SM group compared to HC group (Fig. 8D). The most important is chr3:19,656,072:19,656,166 (loading weight = 0.46) which overlapped with the first intron of *TNFAIP8L2* (TNF alpha induced protein 8 like 2), followed by chr23:19,239,903:19,239,949 (loading weight = 0.38) and chr3:117,128,685:117,128,768 (loading weight = 0.33), which overlapped with the first introns of *CLIC5* (chloride intracellular channel 5) and *LRRFIP1* (LRR binding FLII interacting protein 1), respectively. The dMHB chr3:117,128,685:117,128,768 is also one of the top 10 dMHBs strongly negatively associated with Factor 2. The signature chr3:8,902,020:8,902,136 overlapped with the promoter and first exon of *CD48* (CD48 molecule) harboring the transcription start site. The other dMHB signatures were all overlapped with the first intron of corresponding genes (Table S5). All DEG, DEL and DEM signatures were down-regulated in SM group compared to HC group (Fig. 8E–G). *TMEM53* (transmembrane protein 53), bta-miR-151-3p and LOC107133302 with loading weights 0.51, 0.87 and 0.48, respectively are the most important discriminant signatures from the omics of DEG, DEM and DEL (Fig. 8E–G). Notably, the DEGs *ACOT4* (acyl-CoA thioesterase 4) and *HDDC3* (HD domain containing 3) were also identified as discriminant signatures for *S. aureus* subclinical mastitis in our previous study [15]. The third most important DEM signature, bta-miR-99a-5p, also associated strongly and positively with Factor 1 (weight = 0.71), further revealing its importance in representing the variation of miRNAs during subclinical mastitis. Although the identified DEG, DEL and DEM signatures were strongly correlated, unfortunately, no target relationship was observed amongst them.

## Discussion

A plethora of studies have contributed to the exploration of the genetic architecture underlying bovine mastitis [12, 47–50], however, much of the intricate mechanisms still remain unexplained. One possible reason is that most previous studies considered single data types, such as RNA transcripts [48, 49], DNA sequence variants [51, 52], non-coding RNA transcripts [53, 54], and DNA methylation [18, 55]. These single-data based studies provide limited insights and assembled only fragmented pieces of the puzzle; which does not consider the interconnectedness and synergistic effects that arise from the integration of multiple data layers. Recently, multi-omics approaches that integrate and analyze diverse genomic data sets simultaneously have been identified as imperative to fully unravel the complexities of the molecular mechanisms of disease conditions and the discovery of reliable biomarkers [56–58]. Hence, this study employed different multi-omics approaches to integrate four omics data sets, including the DNA methylation profile and the transcriptomes of mRNA, lncRNA and miRNA, to deepen our understanding of the molecular architecture of subclinical mastitis. To our best knowledge, this study is the first to integrate more than 3 omics data sets to unravel the genomic and epigenomic basis of mastitis. We provide deeper insights into the multi-level signatures simultaneously involved in the regulation of mammary gland response to subclinical mastitis. Our data is consistent with previous studies about bovine mastitis that focused on the integration of two omics data sets [26–28, 30–32, 50] and at the same time provided deeper insights into the molecular signatures of subclinical mastitis.

This study identified the altered activities of biological processes and pathways with the involvement of DNA methylation signatures, genes, lncRNAs and miRNAs, during subclinical mastitis. These multi-omics signatures served as reference for the construction of comprehensive regulatory networks of the crucial biological processes required for mammary gland defense against subclinical mastitis. For example, the “*Staphylococcus aureus* infection” pathway has been identified through many single-omics studies as a pathway of interest for subclinical mastitis caused by *S. aureus* [15, 59]. The integration of epigenomic signatures (dMHBs, DELs and DEMs) with DEGs involved in this pathway were revealed in this study, which could provide more information to better understand the underlying regulatory mechanisms (Fig. 7). For instance, the hypo-methylated dMHB chr18:54,314,202:54,314,226 is found at the regulatory region of the upregulated *C5αR1*, which plays important roles in the inhibition of chemotaxis and phagocyte activation. Moreover, a hypo-methylated dMHB chr16:4,555,707:4,555,760 overlapped with the promoter of up-regulated *IL10*, which is involved in the secretion of cytokine and synthesis of interferon in the inflammation process. The demethylation at regulatory regions has previously been identified as an important regulatory mechanism of the activation of corresponding genes [60–62]. Therefore, the hypo-methylation detected at the regulatory region of these genes (*C5αR1* and *IL10*) may serve as a possible regulatory mechanism of their activation and thereby the corresponding immune processes. Besides, *IL10* was also potentially targeted by 3 differentially expressed non-coding RNAs, including the down-regulated bta-miR-378 and the up-regulated bta-miR-6119p and LOC104974370 (lncRNA) which may post-transcriptionally modulate the expression of *IL10* during subclinical mastitis. Notably, the altered expression of *IL10* has been associated with subclinical mastitis in previous studies [19, 63, 64], highlighting its crucial role in regulating mammary gland inflammation. Therefore, the epigenetic signatures associated with *IL10* may represent the molecular mechanisms regulating its altered expression in response to subclinical mastitis.

Unlike clinical mastitis, where the immune response triggers a visible inflammatory reaction, subclinical mastitis elicits a more subdued and prolonged immune response. The immune system upon recognizing the presence of the pathogen triggers the innate immune response by recruiting immune-related cells, particularly macrophages and neutrophils, into the mammary gland [65, 66]. Consistent with this immune response, many GO terms and KEGG pathways related to leukocyte activities, such as “Leukocyte cell cell adhesion”, “Neutrophil migration”, “Myeloid leukocyte migration”, “Leukocyte mediated immunity” and others (Table S1), were detected with a higher overall active level in subclinical mastitis group compared to healthy control group. However, due to the subdued immune response of the mammary gland and the development of biofilm which provides protection from host immune responses, pathogenic bacteria (e.g., *S. aureus*) are capable of multiplying and colonizing mammary gland tissue leading to long-term infection with no visible symptoms but elevated somatic cell count in milk [67, 68]. Also, when the innate immune response fails to eradicate the invading pathogens, the adaptive immune responses, such as antigen presentation, T cell and B cell activation, and antibody secretion, are activated for further defense of pathogen invasion [69]. Consistent with this, we found that the alterations of multi-omics signatures related to subclinical mastitis were enriched in these processes, such as “T cell activation”, “Regulation of T cell activation”, “Adaptive immune response” and “B cell differentiation” among others (Table S4). It is worth noting that the multi-omics signatures showed concordant contributions to the up-regulation of most immune-related processes and pathways in subclinical mastitis group (Fig. 5). This suggests that the hypo-methylation and enhanced DELs may mediate the up-regulated expression of DEGs thereby contribute to regulate the immune response to subclinical mastitis. The association of epigenetic signatures to these important processes in this study provides a more holistic view of their regulatory networks. For instance, hypo-methylated dMHBs (chr3:8,902,020:8,902,136 and chr3:8,916,776:8,916,809) and the down-regulated DEMs (bta-miR-3431, bta-miR-2285t, and bta-miR-455-3p) may mediate the up-regulated expression of *CD48* which is involved in the communication between target cells and natural killer cell in the “natural killer cell mediated cytotoxicity” pathway (Fig. 6). Moreover, down-regulated bta-miR-143 and bta-miR-205 potentially played roles in the up-regulated expressions of *INFG* and *FAS,* respectively, which are involved in the cytokine-cytokine receptor interaction pathway. Consistent with our results, the altered expression of bta-miR-143 and bta-miR-025 have been previously associated with bovine mastitis [54, 70], further suggesting their regulatory roles in subclinical mastitis. Besides, the abundant hyper-methylated dMHBs at the regulatory region of *PYK2* and multiple DELs targeting *FAS* could potentially open new avenues for investigating the underlying regulatory mechanisms governing the abnormal expression of these genes and thereby the active level of the “natural killer cell mediated cytotoxicity” pathway during subclinical mastitis. In a similar manner, we believe that the involvement of multi-omics signatures could provide valuable information to enhance our understanding of regulatory mechanisms underlying the important biological processes and pathways required for the mammary gland defense against subclinical mastitis.

Furthermore, mammary epithelial cells play a role in defending against invading pathogens causing subclinical mastitis by producing antimicrobial peptides and shedding off infected cells [69, 71]. Consistently, we found that the biological processes related to epithelium development, such as “positive regulation of mammary gland epithelial cell proliferation”, “epithelial cell maturation”, “mammary gland epithelium development” and “epithelial structure maintenance” among others (Table S1) showed general higher activity in subclinical mastitis group according to all multi-omics signatures, which may contribute to the epithelial defense against the invasion of *S. aureus* and *S. chromogenes*. Nevertheless, we also observed the significant involvement of down-regulated DEGs and DELs which negatively associated with Factor 1 and hyper-methylated dMHBs which negatively associated with Factor 2 in processes related to epithelium development, which may explain the impaired mammary gland homeostasis and functions during the long-term course of subclinical mastitis.

Consistently, the overall lower active level of some metabolic processes, such as “fatty acid catabolic process”, “fatty acid derivative metabolic process” and “long chain fatty acid transport”, etc. (Table S1), were observed in cows with subclinical mastitis compared to healthy cows. This disparity in metabolic activity underscores the metabolic alterations that occur in cows experiencing subclinical mastitis. Moreover, some of the metabolic processes related to milk synthesis, such as “fatty acid metabolic process”, “lipid oxidation”, “fatty acid biosynthetic process” were also found to be significantly enriched by down-regulated DEGs that were positively associated with Factor 1 (Table S4A). This intriguing finding suggests a potential interplay between suppressed gene expression and the modulation of these critical pathways, possibly influencing the reduced milk production observed in cows with subclinical mastitis. Adding a nuanced perspective to this scenario, the negative regulation of certain metabolic processes has been found to exhibit relatively higher activity levels in cows with subclinical mastitis, as highlighted by processes like “negative regulation of lipid metabolic process”, “negative regulation of calcium ion transport”, and “negative regulation of fat cell differentiation” (Table S1). These elevated negative regulatory processes could indicate a concerted effort by the cow’s system to counterbalance the metabolic disruptions brought about by the subclinical mastitis condition. Collectively, these observations unveil potential underlying regulatory mechanisms responsible for the compromised milk production performance in cows with subclinical mastitis. The evident altered active level of key metabolic pathways associated with milk synthesis, suggests a complex interplay of molecular events that contribute to the reduced milk production observed in subclinical mastitic cows. By elucidating the involvement of multi-omics signatures in these regulatory dynamics, we may gain a deeper understanding of the genetic and epigenetic basis behind the milk production decline in cows affected by subclinical mastitis, paving the way for targeted interventions and management strategies to mitigate these effects and enhance overall dairy productivity.

Moreover, we report a high number of multi-omics signatures with significant alterations associated with subclinical mastitis, a small subset of which are identified as candidate discriminant signatures of subclinical mastitis. The discriminant signatures described the most variation between subclinical mastitis group and healthy control group and have the potential to be used as biomarkers for improving mastitis control strategies. This study identified 5 Factors driving the principal variance from one or more omics related to subclinical mastitis. Interestingly, the significant signatures associated with Factors 1 and 2 are involved in biological processes and pathways related to the mammary gland response to subclinical mastitis, highlighting the regulatory roles of Factors 1 and 2. For instance, we observed the significant involvement of the up-regulated DEGs and DELs negatively correlated with Factor 1 and hypo-methylated dMHBs negatively associated with Factor 2 in the immune-related processes. In addition, the combination of Factors 1 and 2 clustered the cows into subgroups consistent with their mammary gland health condition (subclinical mastitis or healthy) (Fig. 5M). This indicates that the combination of Factors 1 and 2 has the potential to predict if the cows have subclinical mastitis, and could be used to improve the genomic selection program to further improve dairy cows’ resistance to mastitis. The discriminant signatures—14 dMHBs, 25 DEGs, 18 DELs and 5 DEMs—are highly correlated and able to distinguish healthy cows from sick cows with subclinical mastitis (Fig. 8). Amongst the identified dMHB signatures, the hypo-methylated dMHB chr3:8,902,020:8,902,136 harbored the TSS of up-regulated *CD48*, whose up-regulated expression has also been detected in *E. coli* infected mammary gland tissue [72]. The *CD48* is an important number of the signaling lymphocyte activation molecule family and participates in the adhesion and activation of immune-related cells [73] involved in communications between target cells and natural killer cells in the “natural killer cell mediated cytotoxicity” pathway. DNA methylation near TSS has been revealed to block the expression of corresponding genes [60], therefore, the hypomethylation of chr3:8,902,020:8,902,136 may activate the expression of *CD48* during subclinical mastitis. Other identified dMHBs candidates which overlapped with the first intron of genes (*TNFAIP8L2*, *CLIC5*, *LRRFIP1*, *TMEM229B* (transmembrane protein 229B), *PPP1R12A* (protein phosphatase 1 regulatory subunit 12A), *IDH2* (isocitrate dehydrogenase (NADP(+)) 2), *CORO7* (coronin 7), *USP47* (ubiquitin specific peptidase 47), *PSAP* (prosaposin), *KCTD1* (potassium channel tetramerization domain containing 1), *FNBP1* (formin binding protein 1), *RBMS1* (RNA binding motif single stranded interacting protein 1), and *EHF* (ETS homologous factor)), are being associated with bovine mastitis for the first time. Three discriminant DEG candidates, including *TMEM53*, *ACOT4* and *HDDC3,* have also been identified as discriminant signatures for *S. aureus* subclinical mastitis [15, 34]. Consistent with our finding, *ACOT4* has also been identified as a significant gene for chronic subclinical mastitis in Norwegian Red cattle [74]. The up-regulated expression of *TMEM53* has been identified in the blood of goats with a potential role in small ruminant lentiviruses infection [75]. The involvement of these DEGs in subclinical mastitis or other infectious diseases suggests their possible roles in immune responses and highlights their potential as biomarkers for subclinical mastitis which deserves further investigation. Among the 5 DEM discriminant candidates, bta-miR-99a-5p and bta-miR-499 have been associated with *S. aureus* mammary infection [76, 77], revealing their roles in subclinical mastitis. To the best of our knowledge, the discriminant signatures identified in our study are being associated to bovine mastitis for the first time. However, through our comprehensive multi-omics integration, we have highlighted their potential utility as biomarkers for enhancing mastitis control strategies. These findings warrant further investigation and validation to ascertain their clinical relevance and applicability in improving dairy cow health and management practices.

Furthermore, we acknowledge the limitations of our study and propose potential solutions and further study directions to address these limitations. The limited sample size for certain omics data poses a constraint, potentially impacting statistical power and generalizability. For instance, miRNA profiles were only available for 5 out of the 10 healthy control samples, while other omics datasets included 10 samples for the healthy group. To strengthen the robustness and reproducibility of our findings, our next step is to replicate the study with a larger cohort of cows, incorporating more healthy control cows. By expanding the sample size, we can enhance the reliability and validity of our observations. In addition, while we successfully integrated RNA sequencing data, small RNA sequencing data, and DNA methylation sequencing data from milk somatic cells, the absence of other crucial omics datasets, such as DNA sequence (genome), proteome, metabolome, and microbiome data, limits a comprehensive understanding of subclinical mastitis. To fully unravel the complexities of the disease, future studies that include these additional omics datasets will be indispensable. Furthermore, we recognize the significance of functional studies and validation experiments to bridge the gap between our observational findings and the mechanistic aspects of the immune response to subclinical mastitis. Despite these challenges, our investigation offers valuable insights into the genomic and epigenomic signatures of subclinical mastitis in milk somatic cells. Thus, more extensive studies and functional analyses, are necessary to contribute to a better understanding of the regulatory mechanisms underlying subclinical mastitis and its potential application in dairy cattle management. Our study therefore lays a foundation for future research on the genomic and epigenomic aspects of subclinical mastitis, emphasizing the importance of addressing the limitations and pursuing further investigations to advance our understanding of this important dairy cow disease.

In summary, this study provided a more comprehensive view of the regulatory network underlying bovine subclinical mastitis by using multi-omics approaches. Firstly, the important biological processes and functional pathways with the involvement of multi-omics signatures (genes, lncRNA, miRNAs and DNA methylation) were identified, providing biological insights of subclinical mastitis pathogenesis. Secondly, abundant multi-omics altered signatures (30,846 dMHBs, 2552 DEGs, 1276 DELs and 57 DEMs) were associated to subclinical mastitis. Thirdly, 5 Factors driving the principal variance in multi-omics signatures related to subclinical mastitis were revealed, and in particular, the important roles of Factors 1 and 2 in the regulation of immune defense and impaired mammary gland functions during subclinical mastitis were highlighted. The network of multi-omics signatures were also demonstrated for some important biological processes and pathways to present a relatively more comprehensive view of the regulatory mechanisms underlying subclinical mastitis. Fourthly, few highly correlated candidate discriminant signatures (14 dMHBs, 25 DEGs, 18 DELs and 5 DEMs) were identified for subclinical mastitis, having the potential to be further developed as biomarkers for mastitis control strategies. Hence, the multi-omics integration offered a comprehensive and integrated view of the genomic and epigenomic basis of subclinical mastitis thereby facilitating a deeper understanding of mastitis and identifying candidate biomarkers.

## Conclusion

In conclusion, this study employed a multi-omics approach to unveil the intricate regulatory networks underlying bovine subclinical mastitis. By identifying key biological processes and functional pathways through integration of four omics signatures (genes, lncRNA, miRNAs, and DNA methylation), we gained valuable insights into the pathogenesis of subclinical mastitis. The extensive array of altered signatures associated with subclinical mastitis, spanning dMHBs, DEGs, DELs, and DEMs, further underscored the complexity of the condition. Our findings highlighted 5 principal factors steering the variance in multi-omics signatures linked to subclinical mastitis, with a particular emphasis on Factors 1 and 2 regulating immune defense and impaired mammary gland functions. The comprehensive network of multi-omics signatures presented a holistic view of the regulatory mechanisms governing subclinical mastitis. Moreover, the identification of a select few highly correlated candidate discriminant signatures holds promise for their potential development into biomarkers for mastitis control strategies. Through the integration of multi-omics data, this study provides a deeper understanding of the genomic and epigenomic basis of subclinical mastitis, paving the way for future research and the identification of targeted interventions in mastitis management.

### Supplementary Information

**Additional file 1: Table S1A. **Gene set score per gene set per sample. **Table S1B.** Adjusted p value (false discovery rate, FDR) and *P* value per gene set per sample. 

**Additional file 2: Table S2A. **The differential methylation haplotype blocks identified in cows with subclinical mastitis compared to healthy cows.** Table S2B. **The differentially expressed genes (DEGs) identified in cows with subclinical mastitis compared to healthy cows. **Table S2C. **The differentially expressed long non-coding RNAs (DELs) identified in cows with subclinical mastitis compared to healthy cows. **Table S2D. **The differentially expressed micro RNAs (DEMs) identified in cows with subclinical mastitis compared to healthy cows. 

**Additional file 3: Table S3A.** Factor values of factors in each sample. **Table S3B.** Weight of differentially expressed genes per factor. **Table S3C.** Weight of differentially expressed lncRNAs per factor.** Table S3D.** Weight of differentially expressed miRNAs per factor. **Table S3E.** Weight of differential DNA methylation haplotype blocks per factor.

**Additional file 4: Table S4A.** The gene sets enrichment for differentially expressed genes (DEGs) assocatied with each factor. **Table S4B.** The gene sets enrichment for differentially expressed long non-coding RNAs (DELs) assocatied with each factor.** Table S4C.** The gene sets enrichment for differential methylation haplotype blocks (dMHBs) assocatied with each factor. **Table S4D.** The gene sets enrichment for differentially expressed micro RNAs (DEMs) assocatied with each factor.** Table S4E.** The comparison of gene sets significantly enriched by different omics signatures assocatied with Factors 1 and 2.

**Additional file 5: Table S5. **Candidate discriminant signatures from different omics for distingusing subclinical mastitis from healthy cows.

## Data Availability

The datasets generated and/or analyzed during the current study are available in the NCBI Sequence Read Archive (SRA) under the BioProject: PRJNA962099 and PRJNA962142 (whole genome DNA methylome data), BioProject: PRJNA878880 and PRJNA967255 (RNA-Seq data) and BioProject: PRJNA1021679 and PRJNA1021683 (small RNA-Seq data).

## Abbreviations

ACOT4: Acyl-CoA thioesterase 4
BP-GO: Biological processes gene ontology terms
C5αR1: Complement C5α receptor 1
CD48: CD48 molecule
CD72: CD72 molecule
CLIC5: Chloride intracellular channel 5
CORO7: Coronin 7
CPB2: Carboxypeptidase B2
CSN1S1: Casein alpha-S1
CSN1S2: Casein alpha-S2
CSN2: Casein beta
CXCR1: Chemokine (C-X-C motif) receptor 1
CXCR2: C-X-C motif chemokine receptor 2
DEG: Differentially expressed gene
DEL: Differentially expressed long non-coding RNA
DEM: Differentially expressed microRNA
DIABLO: Data integration analysis for biomarker discovery using a latent component method for omics studies
dMHB: Differential methylation haplotype block
EHF: ETS homologous factor
FAS: Fas cell surface death receptor
FC: Fold change
FCER1G: Fc epsilon receptor Ig
FCGR3A: Fc gamma receptor IIIa
FDR: False discovery rate
FNBP1: Formin binding protein 1
GLYCAM1: Glycosylation dependent cell adhesion molecule 1
GSS: Gene set score
GWAS: Genome-wide association studies
HC: Healthy control
HDDC3: HD domain containing 3
HSTN: Histatherin
IDH2: Isocitrate dehydrogenase (NADP( +)) 2
IFNG: Interferon gamma
IL10: Interleukin 10
ITGAL: Integrin subunit alpha L
ITGB2: Integrin subunit beta 2
KCTD1: Potassium channel tetramerization domain containing 1
KEGG: Kyoto Encyclopedia of Genes and Genomes
LALBA: Lactalbumin alpha
lncRNA: Long non-coding RNA
LRRFIP1: LRR binding FLII interacting protein 1
MHB: Methylation haplotype block
MHL: Methylated haplotype load
miRNA: MicroRNA
MOFA: Multi-Omics Factor Analysis
NAIP: NLR family apoptosis inhibitory protein
ncRNA: Non-coding RNA
PAEP: Progestagen-associated endometrial protein
PPP1R12A: Protein phosphatase 1 regulatory subunit 12A
PSAP: Prosaposin
PYK2: Protein tyrosine kinase 2 beta
RBMS1: RNA binding motif single stranded interacting protein 1
SAP: Tested positive for Staphylococcus aureus
SCC: Somatic cell count
SCP: Tested positive for *Staphylococcus chromogenes*
SEMA4A: Semaphorin 4A
SLC25A19: Solute carrier family 25 member 19
SM: Subclinical mastitis
SM-SAP: Subclinical mastitis tested positive only for *Staphylococcus aureus*
SM-SCP: Subclinical mastitis tested positive only for *Staphylococcus chromogenes*
STEAP4: STEAP4 metalloreductase
TGM3: Transglutaminase 3
THBD: Thrombomodulin
TMEM229B: Transmembrane protein 229B
TMEM53: Transmembrane protein 53
TNFAIP8L2: TNF alpha induced protein 8 like 2
USP47: Ubiquitin specific peptidase 47
WGMS: Whole genome-wide DNA methylation sequencing
